# Factors of decisive importance for being in work or not during two years after breast cancer surgery: content analysis of 462 women’s open answers

**DOI:** 10.1186/s12905-021-01468-1

**Published:** 2021-09-14

**Authors:** Agneta Wennman-Larsen, Veronica Svärd, Kristina Alexanderson, Emilie Friberg

**Affiliations:** 1 Division of Insurance Medicine, Department of Clinical Neuroscience, Karolinska Institutet, 17177 Stockholm, Sweden; 2 Department of Nursing Science, Sophiahemmet University, Box 5605, 11486 Stockholm, Sweden; 3 Department of Social Work, Södertörn University, 14189 Huddinge, Sweden

**Keywords:** Breast cancer, Sick leave, Work, Return-to-work, Sickness absence, Women, Insurance medicine

## Abstract

**Background:**

Paid work is one of the most important aspects in life among working-aged women diagnosed with breast cancer. Despite several attempts, no previous study provides a comprehensive overview from the women’s perspective about factors of importance for being able to work or not. Therefore, the aim of this study was to gain knowledge about factors that women themselves state are of decisive importance for being able to work or not during the first two years after breast cancer surgery.

**Methods:**

Data was collected in a two-year follow-up questionnaire within the frame of a prospective cohort study of working-aged women who had undergone breast cancer surgery. 749 were included in the questionnaire study and of the 616 (82%) responding women, 462 (75%) wrote statements on an open-ended question about factors of decisive importance for being able to work or not work during the past two years. The statements were analyzed with content analysis.

**Results:**

Five categories of factors of importance for being able to  work or not were identified, each covering several sub-categories: Health and wellbeing, Contacts and encounters, Flexibility and adjustment possibilities, Socioeconomic consequences from working/not working, and Own motivation and characteristics. A wide variety of factors were mentioned by the women and the findings give a multifaceted picture of many single but interrelated factors of decisive importance for being able to work/not work. The importance of flexibility in the return-to-work process was stressed, as well as the importance of supportive encounters from, e.g., colleagues, managers, as well as relatives.

**Conclusions:**

The results give a comprehensive overview over a variety of different types of factors for being able to return to/remain in work or to not work after breast cancer surgery, adding new knowledge about e.g. the importance of colleagues, and the women’s own preferences or characteristics. These are factors that different stakeholders, both from healthcare but also from the work place and the insurance office, need to be aware of and collaborate around to support women with breast cancer during the period of treatment, rehabilitation and return to work.

## Background

About 50% of women diagnosed with breast cancer (BC) are of working age and paid work has been shown to be one of the most important aspects in life in these women [1, 2]. Among cancer survivors of working age, paid work not only provides economic security, but also provides normalcy, meaning, and a structure of everyday life and is of importance for identity, self-worth, and a sense of purpose in life [3]. In Sweden, as well as in many other western countries, most women diagnosed with BC are cured from their disease or live with it as a chronic condition. The five-year survival rate is 90%, and the ten-year survival rate over 80% [2, 4]. Nevertheless, studies have shown long-term consequences for employment and sickness absence (SA) [5], and the proportions of women returning to work one year after BC varies significantly between countries with different social insurance systems, from, e.g., 43% in the Netherlands to 98% in the US, while around 60% has been reported in Sweden [6]. Another study from Sweden showed that three years after the BC diagnosis date, 62% had no SA or disability pension [7]. This highlights the importance of gaining in-depth nation-specific knowledge about factors that women themselves perceive to be of importance for their return-to-work (RTW) process or for avoid long-term SA.

Several attempts have been made to understand factors of importance for RTW after BC and in 2018 a systematic review of 26 reviews [8] exploring factors facilitating or impeding RTW, interventions to enhance RTW, lived experiences regarding RTW, and economic aspects related to BC and RTW found that many studies agree on that the factors that facilitate or impede RTW can be categorized into socio-demographic, disease- and treatment-related, as well as psychological, work-related, and policy and economic factors.

Qualitative studies of work-related aspects following a BC diagnosis have also been conducted, but none of these focused on factors of importance for being able to work or not work [8] even if factors related to the RTW-process are identified [9–12].

In addition to reviews in the field of (breast) cancer, theories and models have been published and used as framework in studies about RTW and SA [3, 13–22]. However, Costa-Black et al. states in their text *Work Disability Models: Past and Present* [23] that up until now there has been no single model that covers all aspects of the complex factors that are influencing the individuals’ decisions about work after disease or disability, and only two models have been dealing specifically with the situation of individuals following a cancer diagnosis [16, 20]. In conclusion, even though there are quite many studies about factors of importance for RTW and SA within the field of BC, no single study provides information from the women’s perspective about factors of importance for being able to be in paid work or not, following BC surgery. Therefore, the aim of this study was to gain knowledge about factors that women themselves state are of decisive importance for being able to work or not during the first two years after breast cancer surgery.

## Methods

Data was collected by a two-year follow-up questionnaire within the frame of a prospective cohort study on “Life situation and return to work after breast cancer surgery” [24]. The study base for the cohort study were 970 women who had undergone a first BC surgery in one of three hospitals in Stockholm, Sweden when aged 20–63 years. Two other inclusion criteria were: living in Stockholm County and literate in Swedish. Exclusion criteria were known distant metastases, pre-surgical chemotherapy, and/or a previous BC diagnosis. The women were screened for eligibility consecutively after surgery at their first visit for discussion of further treatment. Inclusion lasted from June 2007 to November 2009. Of the 970 women, 749 agreed to take part in the study and responded to a first comprehensive questionnaire regarding different aspects of SA and (return to) work, which was followed by five additional questionnaires during the following 24-months. The questionnaires were sent home to the women together with a prepaid return envelope; three reminders were sent to non-responders. Thus, the last 24-month follow-up was sent out in 2009–2011 and, 616 women (82.2%) answered the 24-month questionnaire and of those 462 (75%) gave written answers to an open-ended question (described below) and comprise the basis for analysis in this study (Table 1). All methods were carried out in accordance with relevant guidelines and regulations.

**Table 1:** Information of the women initially included and of those who answered the here studied question

	All included at baseline (n = 749)	All who answered the 6th questionnaire at the 24-month follow-up (n = 616)	Answered the open-ended question studied here (n = 462)
Age at inclusion	mean = 51.0, SD = 8	mean = 51.5, SD = 8	mean = 51.0, SD = 8
	n (%)	n (%)	n (%)
University education	410 (55)	350 (57)	286 (62)
Married/cohabitant	406 (54)	348 (57)	263 (57)
*Type of planned treatment*			
Mastectomy	251 (34)	210 (34)	161 (35)
Axillary clearance	286 (38)	227 (37)	180 (39)
Radiotherapy	611 (82)	506 (82)	385 (83)
Endocrine therapy	610 (81)	499 (81)	375 (81)
Chemotherapy	355 (47)	284 (46)	223 (48)
*Working to some extent at*	Inclusion	24-months	24-months
	645 (86)	507 (82)	397 (86)

### Data

The open-ended question that served as basis for the analysis was: If you consider the time since you had your first BC surgery, rank the three most important factors, good and bad, that have been of decisive importance for how you have been able to work/not work. There were three lines, each numbered 1, 2, and 3, respectively—that means that the space for writing statements was very restricted. Thus, the written statements were mostly short, consisting of a few words or up to one sentence, with the most extensive being three sentences. In the analysis, the ranking was not used since many women did not rank their answers, but rather wrote their statements as running text. This open question was placed in the later part of the very comprehensive questionnaire including 388 different questions. The questions before this one in the questionnaire, as well as questions in the five previous questionnaires, concerned different aspects of health, morbidity, healthcare, life situation, life quality, and paid and unpaid work. Thus, the women had already before this question considered many such aspects when responding.

### Analysis

The analysis of the written statements was made with a inductive manifest content analysis [25, 26], using NVivo 9 [27]. An inductive approach was chosen even if the analysis was guided by the researchers’ previous experience from the field, as well as previous models and theories presented [8, 23, 28]. The manifest content analysis refers to the analysis of content that is directly observable in the features of reporting [25]. Since the open-ended question was directly related to the aim of this study, all written statements were considered relevant for the analysis, and 1549 text units were identified from the statements and thus coded. One of the authors (AWL) first coded the whole material of individual responses while another author (EF) independently coded parts of the material. These codes were then discussed in with a third author (VS) and a coding scheme were agreed on that was used for a secondary coding used for developing categories [25]. Thereafter, all authors went through the written statements assigned to specific categories, and if not agreeing, this was discussed until agreement was reached.

### The Swedish context

Women’s prerequisites for RTW are related to the wider context in which they live. From an international perspective, a very high proportion of working-aged women are in paid work in Sweden, also in higher ages [29]. Sweden also has a comparably extensive welfare system, including well-developed and subsidized systems for child care, healthcare, prescribed medications [30, 31], and public social insurances such as SA and disability pension benefits. All individuals 16 years or older with income from work, unemployment, or parental benefits can be granted SA benefits up to 80% of lost income from the Social Insurance Agency (SIA) if having reduced work capacity due to disease or injury. The SA benefits can be granted for several years and for full- or part-time (25%, 50%, 75%, or 100%) of ordinary work hours [32].

## Results

Of the 462 women who wrote statements about factors that hindered or promoted paid work in the two years following their first BC surgery, a somewhat higher proportion had higher education than of all the 749 women who responded to the baseline questionnaire and the 616 women who responded to most other parts of the 24-month follow-up questionnaire (Table 1). There were no differences between the groups concerning primary treatment.

In the analyses of the written statements, five categories were identified covering a wide range of areas, each holding several sub-categories. These five categories were: (A) Health and wellbeing, (B) Contacts and encounters, (C) Flexibility and adjustment possibilities, (D) Socioeconomic consequences from working/not working, and (E) Own motivation and characteristics (Table 2). Some statements had a clear positive or negative relation to being in paid work, yet, it was not always possible to interpret such directions in a conclusive way from the statements. The five categories are presented below in falling order, based on number of statements covered by each category.

A. Health and wellbeing

Almost two fifths of the codes fell in this category, which includes five subcategories covering different factors of health and wellbeing, in general, in relation to the disease and treatment, and from other factors of life.

**Table 2:** Factors of importance for work or not during two years after breast cancer surgery

A. Health and wellbeing	B. Contacts and encounters	C. Flexibility and adjustment possibilities	D. Socioeconomic consequences from working/not working	E. Own motivation and characteristics
A.1. General health and wellbeing	B.1. At the workplace	C.1. Work content, scheduling, and place	D.1. Distraction and normalcy	
A.2. Having had symptoms or problems	B.2. Family, relatives, and friends	C.2. Extent and timing of work or SA	D.2. Economic security or economic stress	
*A.2.a. Mental or emotional symptoms or problems*	B.3. Healthcare	C.3. Treatment and healthcare appointments		
*A.2.b. Physical symptoms or problems*	B.4. Other organizations	C.4. Transportation		
A.3. Consequences related to effects, and timing of treatment				
A.4. Disease prognosis or progress				
A.5. Rehabilitation measures				
A.6. Factors related to other aspects than the BC that may be related to wellbeing				

Categories in letters (e.g., A. or B.), sub-categories in numbers (e.g., A.1. or B.1); Sub-sub-categories in italics (e.g., *A.2.a.)*

1. General health and wellbeing

This sub-category includes more general statements such as good health, wellbeing,  being too ill, or more precise aspects of health such as feeling mentally well/unwell. Some statements emphasized a change in health in relation to before during the disease trajectory, e.g., that they had recovered from the disease but also from the treatment, “have had more energy – managed more” or “I have become stronger, mentally”.

A.2. Having had symptoms or problems

In this subcategory we identified two groups, one regarding a) mental/emotional symptoms or problems and one regarding b) physical symptoms or problems, including statements related to the disease itself, the treatment, or to having sequelae and side effects from the treatment.

- *Mental or emotional symptoms or problems*

In this group, different words were used, such as psychological, mental, and emotional symptoms or problems. The most mentioned were related to fatigue and/or exhaustion, lack of energy or strength. Sometimes this was stated in relation to other factors, e.g., “Short sick-leave period, so I was tired the first weeks” or “the radiation period made me extremely tired—neg”. In some cases, mental problems were mentioned as a consequence from fatigue, e.g., “tiredness = fatigue, exhaustion when doing tough activities resulting in panic disorder”. Related to this, a few women also mentioned insomnia and sleeplessness as important factors. Mental health and/or mental state were also mentioned in more general manners, such as “mental stability” or “had a crisis reaction”. Depression and low spiritedness were mentioned by some women, as were chock, sadness, burnout, worries, stress, and less tolerance for stress. Some women also stated problems with concentration and memory.

A.2.b. *Physical symptoms or problems*

Statements regarding physical and bodily symptoms or problems often concerned physical condition in general, e.g., physical shape or physical health. Others were more specific; “my body does not have the strength as before the cancer treatment”. Pain was the most frequently mentioned physical symptom and was often referred to in general terms such as having pain, ache, or tenderness. Sometimes the pain location was specified, most often from the joints. Others specified when the pain occurred, e.g., “pain when lifting”. A few women mentioned sweats and flashes. Other physical problems or symptoms mentioned were susceptibility to infections, nerve injury, or anemia.

A.3. Consequences related to effects, and timing of treatment

This category includes general statements about effects on wellbeing of the treatment itself but also about wellbeing in relation to timing of treatment. The general statements were related to treatments such as surgery, radiotherapy, chemotherapy, hormone therapy, immunotherapy, or radiation injury. Some statements indicated feeling better after, e.g., chemotherapy or antibody treatment was over, while others described how their treatment affected health or work capacity, e.g., “Some can work during the treatment period, but I could not, due to complications that occurred”. Some referred to how and what treatment they had received, e.g., that the surgery was simple or small and, therefore, did not have implications for work, or that no adjuvant treatments such as chemo- or radiotherapy had been necessary. There were also statements describing how treatment affected their mental health, e.g., “chemo made me slow and stupid”.

A.4. Disease prognosis or progress

In this sub-category, the statements concerned the medical status or experiences related to the prognosis or progress of the BC. Most statements referred to the type of cancer, or size or limitation of the tumor, ranging from having a mild form of cancer to an incurable disease, “that I got this type of cancer, that I was cured and could keep the breasts”.

A.5. Rehabilitation measures

Some women mentioned rehabilitation measures as being of importance. They mentioned having or not having the possibility for rehabilitation measures, the type of rehabilitation measures, or certain rehabilitation providers.

A.6. Factors related to other aspects than the BC that may be related to wellbeing

In this subcategory, most statements concerned health aspects other than the BC, but also factors in life that may be related to general wellbeing, and thus may have an impact on if being able to work or not. Some expressed this in general terms, others were more specific like “Important work, I’m thriving, I have a lot to give but can become tired because of too much cancer. Now my mom also [has cancer]” or “Our spare time is precious now. Newlyweds, work at our summer house, worried husband due to cancer progression and spread”. Some women stated that other health conditions than the BC had hindered them from being in work, “My otherwise bad health is the reason for not being able to work”.

B. Contacts and encounters

Encounters from different stakeholders, such as colleagues, workplace managers, family, friends, and healthcare professionals, or experiences of such encounters were stated and almost one third of the codes fell in this category. Colleagues and workplace managers were most frequently mentioned, followed by family and friends and healthcare professionals and these were stated in positive and supportive terms by most women. Some women also stated encounters without specifying from whom, such as being understood or listened to or “had back-up for some time after the surgery” or just “be listened to”.

1. At the workplace

From the workplace, colleagues were most often mentioned, sometimes just by one word, but also described as supportive, good, or understanding. The possibility for openness and dialogue with the colleagues was also stressed. No one mentioned colleagues in negative terms. Managers or employers were also mostly described as supportive, good, flexible, or understanding. However, here negative experiences were also mentioned, such as having had a bad or un-supportive manager that was showing lack of understanding, and one woman mentioned being bullied. Having to change manager after the BC diagnosis was also experienced as problematic. Some women reported factors regarding the work environment in general terms. Either as a general good, understanding, or supportive atmosphere, but also in terms of flexibility or providing adjustment possibilities, both regarding tasks and work hours. Others mentioned the importance of staying in contact with the workplace as well as the feeling of being welcomed back. Some reported negative experiences such as being deprived tasks, not being given adjustment possibilities, or experiencing a distance to the workplace after being sickness absent. Others stated that support from customers, clients, or from children and parents if working in a nursery or school.

B.2. Family, relatives, and friends

Family, relatives, and friends is another sub-category, by most women these persons were described as supportive, understanding, helpful, or loving. The most frequently mentioned specific person in this subcategory was a husband or a partner but also children and, in some cases, more extended family members. A couple of women wrote that they were working together with their husband and got support and understanding from him. Some stated support from friends. Other positive statements were having found a new partner, having good social contacts, becoming a parent, gaining a better relationship with the children, or becoming a grandparent. However, others described loneliness, lack of support, or negative reactions from the husband, e.g., "[My] husband wants a divorce because our economy has become worse and I do not manage to do and take responsibility for everything at home (bad in the short term, maybe good in the long run)”. One woman also mentioned that support from the family had enabled her to keep working but that this, on the other hand, had resulted in her being constantly tired. Other stressful events in the family that were mentioned were children having a hard time, or that close relatives had become ill or died.

B.3. Healthcare

Most statements about the role of healthcare professionals for being able to work or not were stated in positive terms, even if some statements about encounters from individual professionals were described as negative. Some women described good support from healthcare in general, while others mentioned certain professionals, such as physicians, nurses, or medical social workers. Physicians were most often mentioned as supportive in general and especially in writing sickness absence certificates, while nurses were mentioned as kind or supportive in more general terms. Medical social workers were mostly mentioned as supportive in different phases of the disease even if one woman mentioned a negative contact with a medical social worker. The only other negative examples that were stated concerned a physician who did not ask about the woman´s actual work situation, e.g., heavy lifting, which had worsened her physical state, or the more general statement that healthcare was poor at acknowledging strong reactions, or perhaps afraid of such reactions. Besides the above-mentioned professions, only one other profession was mentioned: a chiropractor helping out with back problems.

B.4. Other organizations

Among other organizations than the above mentioned, work and healthcare, the Swedish Social Insurance Agency (SIA) was most often mentioned, while only two statements mentioned the Employment Office. Most statements about the SIA were positive, some were neutral, and about one third of the statements concerned negative aspects. Positive statements about SIA concerned support, positive encounters, or being flexible. The negative statements concerned the opposite; inflexibility, hassle, or ruining things or routines that were working for the woman. An overall lack of support, without organizational specification, was also mentioned, e.g., “…all the effort needed to find out my rights is demanding” and “Had whished for support in changing job”. Two statements concerned patient organizations: both held general positive experiences from participating in group discussions.

C. Flexibility and adjustments possibilities

The category Flexibility and adjustment possibilities covered several areas in life and the following four subcategories were identified; C.1.Work content, scheduling, and place, C.2. Extent and timing of work or SA, C.3. Treatment and healthcare appointments, and C.4. Transportation and communication. About one fifth of the codes fell in this category.

1. Work content, scheduling, and place

Flexibility regarding work-content, scheduling, and place was the largest subcategory, also including statements about having changed work or workplace, becoming unemployed, or having retired. The possibility to have flexible work schedules or work hours was the most frequently mentioned factor, and some women who were self-employed stated this as positive since being able to decide how and when to work. Others mentioned possibilities to adjust work tasks or content in general, while some specified the possibility to ease physically heavy work, limit contacts with clients or costumers, or reduce stress and amount of work. Flexibility was by most expressed as something that was supported by, e.g., employers, managers, and colleagues, while some also mentioned having been encountered with non-flexibility. Many women stressed the possibility to work from home, at least to some extent, or being able to limit work-related traveling as important factors for being able to work. Another aspect mentioned was if not having the possibility to have a substitute, e.g., making it more difficult to be sickness absent, “My knowledge is unique/cannot easily be substituted”. The nature of specific jobs was also mentioned in having no needs for flexibility or adjustments, often referring to that her work was not physically straining.

C.2. Extent and timing of work or SA

Many women stated different aspects of extent, flexibility, and timing of SA or of resuming work as important for being able to work or not. One factor mentioned was the possibility to reduce work hours by being on part-time SA (e.g., 25%, 50%, or 75% of ordinary work hours) or by combining SA benefits with vacation to get a longer leave. Some women stated that they had continued working part-time after having been on part-time SA during the treatment, while others had been working full-time throughout the period of surgery and treatment, e.g., only taking a week off. The possibility to dispose the time on SA or at work according to ones needs both during, after, or to fit treatment was also stressed, e.g., “the SIA was flexible and allowed me to work when I could”. Some also mentioned sickness certificates written in a way that made it possible for themselves to choose whether, or to what extent, they should work or be sickness absent. Others expressed lack of flexibility or adjustment, e.g., that they wished that they had been able to reduce their work hours. Some stated that they returned to work too early or to a too high extent which sometimes had consequences for their health, “Sick leave. Would have coped better and felt better if having been on part-time SA for a longer time”. One woman stated that she had to make up for the work hours during which she had been absent for treatment.

C.3. Treatment and healthcare appointments

A few statements concerned flexibility or adjustment possibilities regarding healthcare or treatment. Having the time for treatment or physician consultations adjusted to their work hours, e.g., having appointments for radiotherapy adjusted to suit the women’s needs was important for being able to work or not. If this was not possible it hindered work “radiotherapy at irregular hours that were announced late – impossible to combine with the time booking at work”.

C.4. Transportation

A few women mentioned transportation as a factor of importance, e.g., “transportations home-hospital-workplace during radiotherapy”, while others had changed their means of transportation, e.g., from public transport to own car. Further, some women mentioned that they lived close to the hospital or to their work why that such adjustments were not necessary.

D. Socioeconomic consequences from working/not working

This category, socioeconomic consequences from working/not working, covered about a tenth of the codes and included two sub-categories: (1) distraction and normalcy and (2) economic security or economic stress.

1. Distraction and normalcy

The importance of the social aspects of work was stressed, e.g., in terms of that work provided social contacts, fellowship, or belonging. Sometimes this was related to colleagues but also to third part, e.g., customers or the children that one was teaching in school. Some women described work as a distraction from thoughts of the disease, which contributed to a sense of things being normal. Others mentioned the workplace as such, e.g., that they thrived at work or had a safe workplace. Other women focused on the content of their work. Some described their work as fun, that they had an important, stimulating, or satisfying work, or that working made them feel good, e.g., “after the sickness absence it is fun to work again”. However, one woman described negative feelings regarding being left behind others when she returned to work, not feeling good enough any longer.

D.2. Economic security or economic stress

Another factor related to consequences from working was economy. Some just stated economy or salary and some women wrote that they had to work due to poor economy, economic worries, or were dependent on their work income. On the other hand, some women described that they had economic security, e.g., that they were provided for by their husband, that their good economy or salary gave them the opportunity to reduce work hours, or to feel safe.

E. Own motivation or characteristics

In the statement in this category covering almost every tenth of the codes, the women referred to themselves; their own personality and motivation, or their own preferences or approaches regarding work—sometimes described as taking actions in order to accomplish certain goals related to being able to work. The statements regarding own preferences were expressed as the woman’s own desire or wish to work, to RTW, and/or own motivation, e.g., “could start working again due to a strong will to come back”. Contrary, a few statements expressed an own desire or will to work less. Some women described personality characteristics or views of oneself, e.g., positive thinking, a positive view of life, stubbornness, or inner strength. Some expressed that the BC had led to a changed view of life that impacted their preferences or strength of will, e.g., “If possible, I have an even more positive view of life which also makes work more fun”, or “I think more ‘do I want this? Is it important?’”. In some statements the preferences and characteristics were more clearly described as turned into taking actions, often in terms of taking care of oneself regarding, i.e., exercise, food, or rest. Some also described how this was done “[I have] seen to that I was becoming as mobile in the upper body/left arm as before” or “That I ‘listen’ to myself and do not push myself to be ‘a good girl’”.

## Discussion

Of the 616 women answering the two-year follow-up questionnaire, 462 women made statements to the open-ended question about factors of decisive importance for being able to work/not work and more than 1500 codes were identified for analysis from these statements. Five main categories were identified, covering a wide range of areas: Health and wellbeing, Encounters and support, Flexibility and adjustment possibilities, Socioeconomic consequences as factors for working/not working, and Own motivation or characteristics. While some of these factors have been well studied before, both in this population, e.g., health- and treatment-related factors, adjustment possibilities including encounters from different stakeholders such as managers, SIA officers, and healthcare professionals [6, 8–10, 12, 28], and in other cancer populations [11, 33, 34], others are not so well studied, e.g., the importance of colleagues, and the women’s own preferences or characteristics.

What is interesting in the results from the women´s open answers are the comprehensive picture they give over different type of factors. Factors that also may be interdependent for being able to work or not. For example, it was not surprising that factors related to health and wellbeing and to disease and treatment were the most often mentioned factors [8, 16], and the role of fatigue impending RTW has been described before in this population [35]. It is instead notable that few women explicitly mentioned rehabilitation measures as a factor of importance since the aim of rehabilitation measures are to overcome and handle sequelae from the disease and its treatment, that in the long run may help the woman to maintain or regain daily activities such as work. Most cancer rehabilitation measures are focusing on physical activity and symptom management, but tend to lack a focus on outcomes important for function and participation in daily activities [36]. In a review of cancer rehabilitation interventions, Hunter et al. [36] found that aspects of RTW were discussed but these constructs were seldom measured as actual outcomes. In a Cochrane review on interventions for RTW following cancer, de Boer et al. [37] found no studies at all on vocational interventions, and even fewer studies have focused on sustainable work-life beyond RTW in cancer patients [38, 39].

Other mentioned factors that may be related to overcome disease and treatment consequences are flexibility and adjustment possibilities. These were mainly mentioned in relation to the workplace, concerning work content, scheduling, and place. These aspects have previously been described as adjustment latitude; possibilities to (temporarily) adjust work demands to loss of function due to disease [17, 18]. In their Illness Flexibility Model (IFM), Johansson and Lundberg state that adjustment latitude affects the extent to which a loss of function affects an individual's work ability [17]. Many of the women also mentioned the importance of a supportive and flexible work manager/employer; most often in positive terms. This is in line with previous findings showing that most of the women in this cohort, to a high degree experienced adjustment possibilities and support from their employers short after surgery [40]. Some of the women in the present study also mentioned nonsupportive encouters from their managers. Previously it has been stressed that support from managers, and workplace adjustments need to be sufficient to support RTW [12]. In the present study, it is also notable that colleagues were the persons most often mentioned as being of importance for being able to work, described as supportive, good, or understanding. More in-depth studies are needed to understand how this works. Colleagues may have an important role both in adjusting the work situation, even if not having such responsibilities, and in providing social contacts, distraction, and support to ease both the physical and mental consequences of the BC. The importance of supportive colleagues for work as well as for vocational satisfaction has also been shown in results from the close-ended questions in one of our previous studies from this cohort [41] as well as in other studies [8, 42, 43], while lack of support from colleagues has been shown to limit and delay RTW [44]. Our results also indicate the importance of having contact with the workplace during SA and to have an open dialogue with colleagues, which is also stated in some previous studies [42, 45, 46]. Support from colleagues is, however, dependent on whether the woman chose to disclose her BC diagnosis at work or not. Disclosure of the diagnosis has been shown to facilitate an open dialogue helping to enable support, while negative experiences have been found in terms of, e.g., receiving unwanted questions or being fired [47]. As the role of colleagues for women’s possibility to work during and following BC seems to be of high importance, this should be further studied.

Statements about flexibility and adjustment possibilities was, however, not only found in relation to the workplace but also in relation to other stakeholders, and the flexibility or adjustment possibilities provided by different stakeholder’s may be interdependent. If, e.g., the treatment appointments are adjusted to working hours, then working hours do not need to be adjusted to treatment appointments, even if adjustment or flexibility possibilities from health care in terms of, e.g., treatment or appointments, seldom were mentioned by the women.

It has been stated in, e.g., in the Cancer and work model [16] that understanding RTW after cancer is complex and that personal characteristics interact with the workplace, the healthcare, and the social security system, as well as economic and legislative conditions but that the interplay between these aspects is hard to capture in empirical studies and need more in-depth studies.

To facilitate the possibility to work during and after treatment, many women also stressed the importance of flexibility regarding the extent and timing of work and/or SA. The possibility in Sweden to be on SA for part-time is rather unique. The possibility to gradually increase work hours, as well as adjusting the timing of this to one’s own situation was stressed as important for being able to work or not. Despite Sweden’s comparably extensive social insurance system, granting SA benefits up to 80% of lost income if having reduced work capacity due to injury or disease [32], some women stated that they were dependent on their income from work. This may have influenced the possibility to gradually increase work hours or to, if needed, be sickness absent for recovery.

Another important factor for being able to work or not as mentioned by the women were their own motivation or characteristics, where they described themselves as taking actions through, e.g., exercise, ‘healthy food’, and rest. The importance of internal and motivational factors in the RTW process has been shown previously [48] where level of motivation is associated with RTW after cancer [49]. That women with BC are actors in their own pace back to work has been described in one of our previous studies based on data from focus groups with women from this cohort early after diagnosis [50]. Thus, it is imperative that healthcare encounters, irrespective of from what profession, does not work against women’s motivation to RTW. The findings showing that internal and motivational factors, as well as colleagues and work are important for women’s rehabilitation and RTW should therefore be acknowledged by healthcare professionals.

### Strengths and limitations

A strength of this study is the large number of participants who answered the question about factors they experienced as being of importance for them to be work or not. The short statements did not always allow a deeper understanding of, e.g., how the factors are related or in what way they are considered important. Still the large number of statements in various fields is a strength of the study showing the complexity of what is of importance for being able to work or not work following breast cancer. Another strength is that the study is conducted in Sweden with a very high female employment rate, also in higher ages [29], meaning that, the health selection into staying in paid work is of less importance. In the overall prospective project, the response rate was nearly 80% despite the very comprehensive questionnaires, indicating that the women considered these topics to be of great importance. According to a previous study from this cohort, the proportion of women rating work as one of the most important aspects of life only decreased from 65 to 60% during the two-year follow-up [41]. Another strength is the multi-professional and inter-disciplinary project group analyzing the data from different perspectives and the use of NVivo [27] in the analyses to enhance good transparency. A limitation may be difficulties for the women to in retrospect remember all relevant factors during the two years that were of importance. This may, on the other hand, also be a strength since when looking back in retrospect, it may be possible to sort out the most important factors. Another limitation is the phrasing of the open-ended question that did not differentiate between hindering or promoting factors. However, since the question cover the whole two-year period from surgery a differentiation could have made it hard for the women to do so since at one point in the disease trajectory an aspect might be experienced as promoting while at another point the same aspect might be experienced as hindering e.g., a certain encounter.

## Conclusion

The results give a comprehensive overview over a variety of different types of factors for being able to return to/remain in work or to not work for women of working ages during the two years after a first breast cancer surgery. Some known before, and some not so well studied, e.g., the importance of colleagues and the women’s own preferences or characteristics. The results may facilitate the support for women after breast cancer from different stakeholders that are encountering them during the period of treatment and rehabilitation measures. Further studies are needed to capture the complexity of the interplay between these factors.

## Data Availability

The data in this project are not publicly available due the General Data Protection Regulation, the Swedish law SFS 2018:218, the Swedish Data Protection Act, the Swedish Ethical Review Act, and the Public Access to Information and Secrecy Act. Readers may contact Professor Kristina Alexanderson (kristina.alexanderson@ki.se) regarding the data.

## Abbreviations

BC: Breast cancer
SIA: Social Insurance Agency
SA: Sickness absence
RTW: Return to work
